# Sensitivity and specificity of detection methods for erythropoietin doping in cyclists

**DOI:** 10.1002/dta.2665

**Published:** 2019-07-17

**Authors:** Jules A.A.C. Heuberger, Peter van Eenoo, Joris I. Rotmans, Pim Gal, Frederik E. Stuurman, Titiaan E. Post, Johannes M.A. Daniels, Herman Ram, Olivier de Hon, Jacobus Burggraaf, Adam F. Cohen

**Affiliations:** ^1^ Centre for Human Drug Research Leiden Netherlands; ^2^ DoCoLab Ghent Belgium; ^3^ Department of Internal Medicine Leiden University Medical Centre Leiden Netherlands; ^4^ Leiden Academic Centre for Drug Research Leiden Netherlands; ^5^ Department of Pulmonary Diseases VU University Medical Centre Amsterdam Netherlands; ^6^ Anti‐Doping Authority the Netherlands, Capelle aan den IJssel Netherlands

**Keywords:** athlete biological passport, doping, erythropoietin, isoelectric focusing, sarcosyl‐PAGE

## Abstract

Recombinant human erythropoietin (rHuEPO) is used as doping a substance. Anti‐doping efforts include urine and blood testing and monitoring the athlete biological passport (ABP). As data on the performance of these methods are incomplete, this study aimed to evaluate the performance of two common urine assays and the ABP. In a randomized, double‐blinded, placebo‐controlled trial, 48 trained cyclists received a mean dose of 6000 IU rHuEPO (epoetin β) or placebo by weekly injection for eight weeks. Seven timed urine and blood samples were collected per subject. Urine samples were analyzed by sarcosyl‐PAGE and isoelectric focusing methods in the accredited DoCoLab in Ghent. A selection of samples, including any with false presumptive findings, underwent a second sarcosyl‐PAGE confirmation analysis. Hematological parameters were used to construct a module similar to the ABP and analyzed by two evaluators from an Athlete Passport Management Unit. Sensitivity of the sarcosyl‐PAGE and isoelectric focusing assays for the detection of erythropoietin abuse were 63.8% and 58.6%, respectively, with a false presumptive finding rate of 4.3% and 6%. None of the false presumptive findings tested positive in the confirmation analysis. Sensitivity was highest between 2 and 6 days after dosing, and dropped rapidly outside this window. Sensitivity of the ABP was 91.3%. Specificity of the urine assays was high; however, the detection window of rHuEPO was narrow, leading to questionable sensitivity. The ABP, integrating longitudinal data, is more sensitive, but there are still subjects that evade detection. Combining these methods might improve performance, but will not resolve all observed shortcomings.

## Graphical abstract

World Anti‐Doping Agency methods for detection of recombinant human erythropoietin were evaluated in cyclists. Detection in urine by sarcosyl‐PAGE had a sensitivity of 63.8% and isoelectric focusing of 58.6%, sensitivity peaked between 2 and 6 days after dosing, rapidly dropping outside this window. False presumptive finding rates were 4.3% and 6%, respectively, but none of the false presumptive findings tested positive in the confirmation analysis. Sensitivity of the athlete biological passport that integrates longitudinal hematological data was 91.3%.

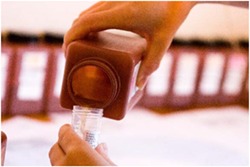

## 1 INTRODUCTION

Athletes have used drugs to enhance sports performance throughout history, despite all efforts to ban these doping substances. To detect cheating athletes, the World Anti‐Doping Agency (WADA) oversees a global doping control system using blood and urine samples that are collected both in competition and out of competition. If an athlete is tested positive, this will usually lead to a suspension of two or four years, depending on the substance found and the circumstances of the case. It can be up to lifetime ineligibility for recidivists.^1^ One substance that is widely abused, especially in endurance sports, is recombinant human erythropoietin (rHuEPO). rHuEPO has shown to positively affect maximal performance,^2^, ^3^ and although there is a lack of clear scientific proof whether it also improves actual endurance performance,^4^ the belief in erythropoietin's effects on performance among athletes, their staff and the general public is overwhelming. Recent confessions to rHuEPO misuse by many cyclists from the 1990–2010 era, including former champions like Lance Armstrong, unfolded an rHuEPO epidemic, at least within cycling. Testing for rHuEPO use was introduced in 2000, and results from the testing figures published by WADA seem to indicate that rHuEPO is still used by athletes.^5^ In the period 2012–2017,^5^ a total of 200 451 urine samples were tested for presence of erythropoiesis stimulating agents (ESAs) and 305 samples (0.15%) produced adverse analytical findings (AAFs). In addition, 12 966 blood samples were tested, of which 61 samples (0.44%) produced AAFs. These AAFs are interpreted as proof an athlete used an ESA. In addition to these specific tests for rHuEPO, WADA introduced the athlete biological passport (ABP) in 2009, in which several biomarkers of an individual are measured longitudinally. The hematological module containing hematological markers is applied to monitor use of erythropoiesis stimulating agents such as rHuEPO. Abnormalities in these markers can be used to start targeted testing in that individual, or even as a doping violation by itself.

Although doping tests are intended to discourage athletes from using doping, detect the ones that are violating doping rules, and protect the athletes that play fair, their ability to do so completely depends on the performance of the tests and an intelligent testing program collecting samples within the window of detection. Technical documents describing the urine assays for detecting ESA use are available through WADA^6^ and articles on the methods have been published.^7^, ^8^, ^9^, ^10^, ^11^ Some information on detection windows for isoelectric focusing (IEF) and sodium dodecyl sulphate polyacrylamide gel electrophoresis (SDS‐PAGE) is available for several rHuEPO variants from open label studies.^12^, ^13^, ^14^ However, well‐designed blinded studies describing the critical assay characteristics of sensitivity and specificity and the detection window, are not sufficiently (publicly) available.^15^ We believe that scientific evidence underpinning internationally and widely applied doping tests should be available in order to understand the value of the tests, protect fair‐playing athletes, and collaborate on improving doping detection. For that reason, we aimed to evaluate the performance of three main methods being used in rHuEPO detection by determining sensitivity and specificity of the sarcosyl‐PAGE and IEF assays on urine samples, and of the ABP and its hematological module.

## 2 METHODS

### 2.1 Study design and participants

For the purpose of this study, blood and urine samples were used that were obtained in a double‐blind, randomized, placebo‐controlled, parallel, single‐center study on rHuEPO, in which 48 healthy male cyclists between 18 and 50 years of age were included. The design of this trial was described elsewhere.^4^ In short, the main inclusion criteria were maximum power‐to‐weight ratio during the maximal exercise test at screening exceeding 4 W/kg, Hb between 8.0 and 9.8 mmol/L (equivalent to 12.8–15.7 g/dL) and Ht below 48% at screening and not being subject to anti‐doping regulation or using medication that could potentially interact with the study drugs or study assessments. All participants gave written informed consent prior to any study‐related activity. The study was approved by the Independent Ethics Committee of the Foundation ‘Evaluation of Ethics in Biomedical Research’ (Stichting Beoordeling Ethiek Biomedisch Onderzoek), Assen, Netherlands. The study is registered in the Dutch Trial Registry (Nederlands Trial Register, NTR) under study number NTR5643.

### 2.2 Randomization and masking

Randomization to the rHuEPO or the placebo group (1:1) was done by a randomization code generated by an unblinded statistician who was otherwise not involved in the execution of the study. Enrolment of subjects was performed by a blinded study physician.

## 3 PROCEDURES

### 3.1 Treatment

Epoetin β (NeoRecormon, Roche, Basel, Switzerland), prepared from multidose vials containing a lyophilizate of 50 000 IU epoetin β and 10 mL solvent for solution for injection, or saline (0.9% NaCl) was administered subcutaneously with weekly abdominal injections for 8 weeks. Dosing aimed to reach a target Hb of 10%–15% increase compared to the baseline Hb value, similar to previous studies investigating effects of rHuEPO on performance.^16^ The first four injections contained 5000 IU per injection. The dose was modulated by an unblinded, non‐study‐related physician to 6000 IU, 8000 IU, or 10 000 IU in the final four weeks in case the Hb level was below the target range, doses similar to known practices in professional cycling.^17^ When the Hb was in the target range, 2000 IU was administered. If the Ht was equal to or exceeded 52% or if the Hb exceeded the upper limit of the Hb range (increase of 15% compared to baseline), a placebo injection was administered.

All participants also received open‐label daily oral doses of 200 mg ferrous fumarate and 50 mg ascorbic acid (both Pharmachemie B.V., Haarlem, Netherlands).

### 3.2 Urine and blood samples for doping detection

Urine samples were collected for each subject and 50 mL was stored in Falcon tubes (Greiner Bio‐One International GmbH, Kremsmuenster, Austria) at −70°C. Samples were collected at approximately 1 hour, 2 days, 4 days, and 7 days after the second dose and at approximately 12 days after the last dose. These samples were taken while subjects had not exercised in the preceding hours. To evaluate potential effects of exercise on the outcome of the test, a sample was taken as soon as the subject could urinate after the maximal exercise at approximately 4 days after the second dose and after a race to the top of Mont Ventoux at approximately 12 days after the last dose. Samples were shipped to the WADA‐accredited anti‐doping laboratory DoCoLab UGent by courier in two separate batches. The lab was aware these samples were research samples, but was blinded to the administered treatment.

All hematology samples were drawn after participants were seated with their feet on the floor for at least 10 minutes, at the following time points: 1–6 weeks before the first dose; directly before the first, third, fifth and seventh doses; approximately 12 days and 4 weeks after the last dose. In general, this means there were 2 weeks between two samples. Samples were analyzed within 2 hours at the Leiden University Medical Centre (LUMC) on a Sysmex XN9000 analyzer (Sysmex Corporation, Kobe, Japan). Samples taken at the Mont Ventoux race were split into two aliquots, one of which was kept at room temperature for determination of leucocytes and leucocytes differentiation and one was kept between 2 and 8°C for determination of hemoglobin, hematocrit, erythrocytes, reticulocytes, MCV, MCH, MCHC, thrombocytes, immature reticulocyte fraction (IRF), and red blood cell distribution width (RDW‐SD). Samples were driven by courier to the LUMC and analysis took place within 36 hours of collection.

### 3.3 Sarcosyl‐PAGE urine assay

The sarcosyl‐PAGE urine assay was used as described in the WADA technical document.^6^ In short, immunopurification of the sample was performed with antibodies other than the one used for immunoblotting before loading 15 μL of sample on pre‐cast polyacrylamide gels (NuPAGE BisTris gels, 10% T, 1.5 mm, as described previously^18^), with 15 slots for test samples per gel (performance conditions: *I* = 1.56 mA/cm^2^ at 0.16A; time = 45 minutes). For purification, Stemcell ELISA was used as described previously, with 15 μL SAR buffer.^11^

In addition, a negative control sample, a positive control sample containing rHuEPO, and a reference to enable to define apparent molecular mass were run in separate slots. Running buffer used was sodium N‐lauroyl sarcosinate, and after the electrophoretic separation double‐blotting was performed with the monoclonal mouse anti‐human EPO antibody clone AE7A5 and a secondary antibody. The secondary antibody was a goat anti‐mouse biotinylated polyclonal antibody IgG (H + L), cross‐adsorbed, HRP (Thermo Scientific product no. 31432) and used in combination with a streptavidin horseradish peroxidase complex (Biospa, Milan, Italy) and a substrate (SuperSignal West Femto Maximum Sensitivity; Thermo Scientific, Waltham, MA, USA). The electrophoretic patterns of ESAs were revealed by the use of an amplified chemiluminescent system. Detection of exogenous EPO, in this case epoetin β, was done on the basis of the characteristic band shape (“broad band”) and different (higher) apparent molecular mass than endogenous EPO. A sample was termed positive if a single band was visible above the negative control EPO, or if a mixed band both at the endogenous and exogenous location or a diffuse or faint area of the band above the corresponding endogenous band were visible, as per the WADA technical document.^6^

### 3.4 Isoelectric focusing urine assay

The IEF urine assay was used as described in the WADA technical document.^6^ In short, immunopurification of the sample was performed with antibodies other than the one used for immunoblotting before loading 20 μL of sample on the gel, with 30 slots for test samples per gel. For purification, Stemcell ELISA was used as described previously, elution with 30 μL 4.4% CHAPS.^19^ IEF was performed in a pH range compatible with the isoelectric points (pI) of both the natural EPO and its recombinant analogues. IEF was performed under denaturing conditions (approximately 7 M urea). After the electrophoretic separation double‐blotting was performed with the monoclonal mouse anti‐human EPO antibody clone AE7A5 and a secondary antibody. The secondary antibody was a goat anti‐mouse biotinylated polyclonal antibody IgG (H + L), cross‐adsorbed, HRP (Thermo Scientific product no. 31432) and used in combination with a streptavidin horseradish peroxidase complex (Biospa, Milan, Italy) and a substrate (SuperSignal West Femto Maximum Sensitivity; Thermo Scientific, Waltham, MA, USA). The electrophoretic patterns of ESAs were revealed by the use of an amplified chemiluminescent system. Detection of exogenous EPO, in this case epoetin β, was done on the basis of the relative location of the bands compared to the position of the bands corresponding to the rHuEPO biological reference preparation (BRP) of the European Pharmacopeia (equimolar mixture of epoetin‐alfa and ‐β), which define the basic and acidic areas. A sample was termed positive if there were at least three acceptable, consecutive bands in the basic area, and if the two most intense bands measured by densitometry were in the basic area, as per the WADA technical document.^6^ It should be noted that the IEF assay is currently not used in routine practice by the DoCoLab and therefore the associated analyses are exploratory.

### 3.5 Urine sample evaluation (screening)

Samples were designated as screening negative, screening positive or non‐detectable by the DoCoLab in Ghent. Samples were non‐detectable if no band was detected in the EPO (endogenous or exogenous) designated region.

### 3.6 Confirmation analysis

After results of the screening analysis were available, the outcome was analyzed by unblinded Centre for Human Drug Research (CHDR) researchers. The WADA procedure describes that samples returning suspicious results at this screening stage are termed “presumptive adverse analytical findings”, and need to undergo confirmation in a confirmation procedure.^20^ In the current study, slightly different terminology was used to differentiate between suspicious results from a subject treated with rHuEPO or with a placebo. Therefore, samples from placebo‐treated subjects that generated a suspicious result in the screening analysis were termed “false presumptive findings”. For the sarcosyl‐PAGE assay these were re‐analyzed with a confirmatory sarcosyl‐PAGE assay. Both the initial and the confirmation methodology were the same. In order to keep this confirmation analysis blinded to the DoCoLab, true positive, true negative, and false negative samples were included as well, and this selection of samples was relabeled by CHDR staff for analysis. Samples were only deemed positive, similar to routine analysis, when they would be forwarded for a mandatory second opinion. The WADA technical document requires^6^ that all presumptive adverse analytical findings are evaluated by a recognized expert. After evaluation by the laboratory performing the analysis, samples are therefore forwarded to a WADA‐recognized expert. Currently, these experts are co‐authors of the WADA technical document^6^ and have extensive scientific knowledge of ESAs, their detection methodology and potential issues in the detection of ESAs. In the framework of this study, samples were not subjected to a second opinion and the interpretation was solely based on the laboratory performing the analysis. No WADA‐recognized expert as per the technical document^6^ was involved. In case of doubt, samples were deemed negative.

### 3.7 Osmolality

Osmolality was determined by taking an aliquot from the homogenized, centrifuged samples, using the freezing point method on an Osmometer Auto & Stat, OM‐6050 (Arkray, Kyoto, Japan). Osmolality determination was initiated after all analyses had been performed to provide insights into potential effects of osmolality on assay results.

### 3.8 Athlete biological passport

A method similar to the adaptive model of the ABP was constructed internally using Microsoft Excel 2013 (Redmond, WA, USA), as the official WADA software was not made available for this study. Individual curves of each separate hematological parameter from blood samples were produced by plotting the observed value, and the 99.7% confidence interval (CI) around that value. For the first measurement, the 99.7% CI was based on the population mean (Pop. mean) ± 3xSD of all available data points from the screening and baseline hematological samples of all subjects that completed these visits (Pop. SD); for IRF and RDW‐SD only the value at baseline was used as they were not determined at screening. For following data points, with each additional available measurement the 99.7% CI was based more on individual data, and calculated as follows:

- second data point:

$$\frac{\left(\mathrm{Pop}.\text{mean}+\text{individual value}\;1\right)}{2}\pm \mathrm{SD}\left[\mathrm{Pop}.\text{mean}+\text{individual value}\;1\right]+2*\mathrm{Pop}.\mathrm{SD}$$

- third data point:

$$\frac{\left(\mathrm{Pop}.\text{mean}+\text{individual value}\;1\;\text{and}\;2\right)}{3}\pm 2*\mathrm{SD}\left[\mathrm{Pop}.\text{mean}+\text{individual value}\;1\;\text{and}\;2\right]+\mathrm{Pop}.\mathrm{SD}$$

- fourth data point:

$$\frac{\left(\mathrm{Pop}.\text{mean}+\text{individual value}\;1,2\;\text{and}\;3\right)}{4}\pm 3*\mathrm{SD}\left[\mathrm{Pop}.\text{mean}+\text{individual value}\;1,2\;\text{and}\;3\right]$$

- fifth data point:

$$\frac{\left(\mathrm{Pop}.\text{mean}+\text{individual value}\;1,2,3\;\text{and}\;4\right)}{5}\pm 3*\mathrm{SD}\left[\mathrm{Pop}.\text{mean}+\text{individual value}\;1,2,3\;\text{and}\;4\right]$$

- etc.

The OFF score (OFFS) was calculated using the following formula: ([hemoglobin concentration in g/dL]) * 10 ‐ (60 * √([Reticulocyte percentage])). Graphs of each separate variable belonging to one subject were combined into one document and sent with a coded (blinded) identifier to the DoCoLab in Ghent for evaluation of a subject being negative, suspicious or positive. Two independent researchers at the DoCoLab, which is designated as an Athlete Passport Management Unit (APMU) by WADA, independently evaluated the data and two sets of results were generated and analyzed, so that reproducibility could also be evaluated.

### 3.9 Exercise

Two urine samples per subject were collected after exercise: one after a maximal exercise test to exhaustion at 4 days (±1 day) after the second dose administration and one approximately 12 days after the last dose after participants competitively completed an uphill road race on the Mont Ventoux. This race was directly preceded by 110 km cycling.

### 3.10 Data management

All data were stored in a clinical trial database (Promasys, Omnicomm Inc., Fort Lauderdale, USA) and checked for accuracy and completeness. A blinded data review was performed before code‐breaking and analysis according to a standard procedure at our unit.

## 4 STATISTICAL ANALYSIS

Standard assay characteristics were calculated based on the reported results. For determining overall urine assay characteristics, only samples that were designated negative or positive were included in the analysis. When determining sensitivity over time, two analyses were done, one including and one excluding the samples designated non‐detectable. For this analysis, samples were binned per day, and only bins with more than one sample were included in the analysis. When determining ABP assay characteristics, suspicious and positive were combined as being an indication of rHuEPO use.

## 5 ROLE OF THE FUNDING SOURCE

This was an investigator initiated study by the foundation CHDR in Leiden in collaboration with DoCoLab (Ghent, Belgium), Leiden University Medical Centre, the Anti‐Doping Authority of The Netherlands and the Department of Pulmonary Diseases, VU University Medical Centre, Amsterdam. There was no external funding source.

## 6 RESULTS

Forty‐nine participants were recruited. One participant withdrew after the first dose administration and was replaced by a reserve participant, whereas another participant withdrew after the fourth dose administration. Both withdrawals were for personal reasons and not related to the study treatment or medical concerns. All 48 subjects were included in the urine detection analysis. Due to the incomplete ABP profile of the withdrawn subject, 47 subjects were included in the analyses for the ABP, of which 23 were in the rHuEPO group and 24 in the placebo group. All participants were living at sea level and did not spend any substantial amount of time at (simulated) high altitude.

Participants in the rHuEPO group received eight dosages of epoetin β during the study. Mean rHuEPO dose was 5000 IU per week during the first 4 weeks of the study and 7000 IU in the subsequent 4 weeks. On five occasions a placebo injection was intentionally administered to subjects in the rHuEPO group, because of the measured hematocrit or hemoglobin levels. Of all 336 urine samples, 6 were not collected due to withdrawal from the study or subjects not being able to perform the particular visit. One additional sample from an rHuEPO treated subject was not analyzed with the IEF assay as it was erroneously not included in the sample shipment at the time of the IEF analysis.

### 6.1 Urine analysis

### 6.2 Sarcosyl‐PAGE urine assay

A total of 330 urine samples were analyzed for rHuEPO using sarcosyl‐PAGE, of which 17 samples (5%) were termed non‐detectable. Of these, 14 (82%) belonged to subjects in the rHuEPO group and 3 (17%) to subjects in the placebo group. Based on the remaining 313 samples, the sensitivity of sarcosyl‐PAGE after the screening analysis was 63.5% and specificity was 95.7% (with seven false presumptive findings; Table 1). These false presumptive findings belonged to urine samples from six subjects.

**Table 1:** Sarcosyl‐PAGE urine assay performance characteristics (total *N* = 330)

**Measure**	**Screening Analysis**	**Screening +Confirmation Analysis**
	**Value (%)**	**Value (%)**
Sensitivity	63.8	63.8
Specificity	95.7	100.0
False negative rate	36.2	36.2
False positive rate	4.3	0.0
Precision	93.1	100.0
False discovery rate	6.9	0.0
False omission rate	25.6	24.8
Negative predictive value	74.4	75.2

Fourteen samples were analyzed for a second time in the confirmation analysis, including all seven samples producing a false presumptive finding in the screening analysis. All 14 samples were correctly identified as negative in placebo subjects and positive for rHuEPO subjects. The assay characteristics when taking both analyses into account show specificity increased to 100% (Table 1).

When comparing samples that were collected before and directly after a maximal exercise test on the same day, sensitivity was 100% at 4 days, 92% and 100% at 5 days, 57% and 0% at 11 days, 33% and 0% at 12 days, and 0% at 13 days since last dosing.

### 6.3 Sarcosyl‐PAGE sensitivity over time

**Figure 1:**
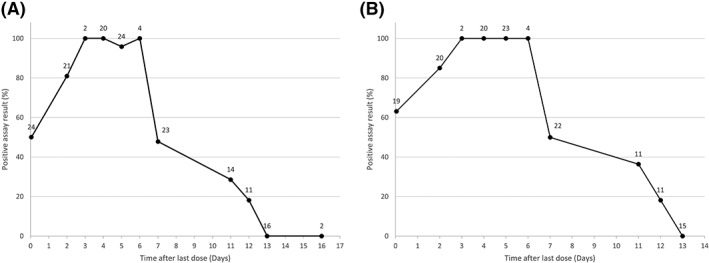
Sarcosyl‐PAGE sensitivity versus time since dose. Sensitivity for the sarcosyl‐PAGE assay over time in the rHuEPO‐treated subjects. The numbers at each data point represent the number of samples that are in a bin. A, includes samples that were designated non‐detectable; B, excludes these samples.

When tested approximately 1–2 hours after the second rHuEPO dosing (and 7 days after the first), 50% of rHuEPO treated subjects tested positive (Figure 1). The sensitivity increased to approximately 81% when urine was collected 2 days after the last dose, and was 95%–100% between day 3 and 6 after the last dose. After 7 days, sensitivity decreased again to approximately 50% and was 29% at 11 days, 18% at 12 days, and 0% at 13 and 16 days after the last dose. When samples designated as non‐detectable are excluded, sensitivity slightly increases, but is very similar.

### 6.4 Isoelectric focusing urine assay

A total of 329 urine samples were analyzed for rHuEPO using IEF, of which 68 samples (21%) could not be declared either positive or negative. Of these, 51 (75%) belonged to subjects in the rHuEPO group and 17 (25%) to subject in the placebo group. Based on the remaining 261 samples, the sensitivity of IEF after a single analysis was 58.6% and specificity was 94.0% (with 9 false presumptive findings; Table 2). These false presumptive findings belonged to urine samples from five subjects (one subject with three false presumptive samples and two with two). There was no overlap with the false presumptive findings in the sarcosyl‐PAGE assay. For IEF, no confirmation analysis was performed. The focus of the urine analysis was on the sarcosyl‐PAGE assay as that is the standard assay at the DoCoLab in Ghent, whereas the IEF assay was only implemented for this study. For this reason we decided not to perform a confirmation analysis for this method. When comparing samples that were collected before and directly after a maximal exercise test on the same day, sensitivity was 50% and 89% at 4 days, 33% and 83% at 5 days, 42% and 0% at 11 days, and 0% at for all samples at 12 and 13 days since last dosing.

**Table 2:** Isoelectric focusing urine assay performance characteristics (total *N* = 329)

**Measure**	**Value (%)**
Sensitivity	58.6
Specificity	94.0
False negative rate	41.4
False positive rate	6.0
Precision	87.8
False discovery rate	12.2
False omission rate	24.6
Negative predictive value	75.4

### 6.5 Isoelectric focusing sensitivity over time

**Figure 2:**
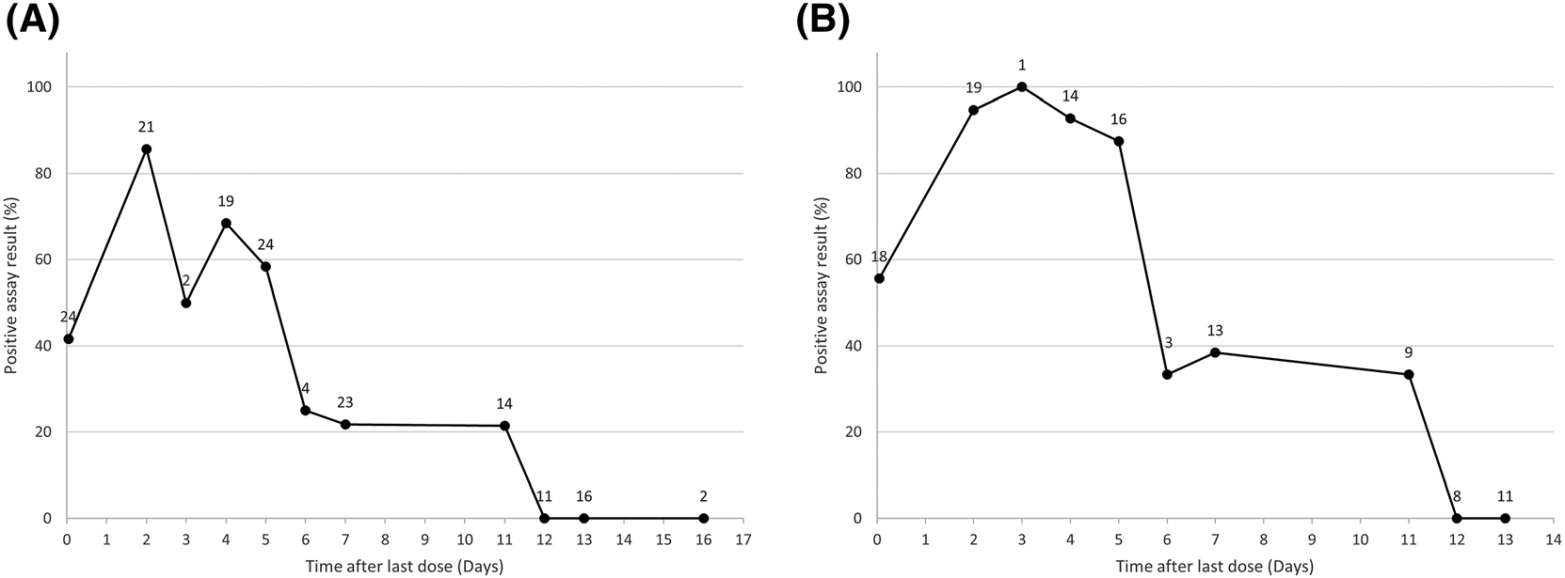
Isoelectric focusing sensitivity versus time since dose. Sensitivity for the isoelectric focusing assay over time in the rHuEPO‐treated subjects. The numbers at each data point represent the number of samples that are in a bin. A, includes samples that were designated non‐detectable; B, excludes these samples.

When tested approximately 1–2 hours after the second rHuEPO dosing (and 7 days after the first), 42% of rHuEPO‐treated subjects tested positive (Figure 2). The sensitivity increased to approximately 86% when urine was collected 2 days after the last dose, and was 50%–68% between days 3 and 5 after the last dose. After 6 and 7 days, sensitivity decreased again to approximately 25 and 22% and was only 21% at 11 days and 0% at 12, 13, and 16 days after the last dose. When samples designated as non‐detectable are excluded, sensitivity increases significantly (Figure 2).

### 6.6 Athlete biological passport

**Figure 3:**
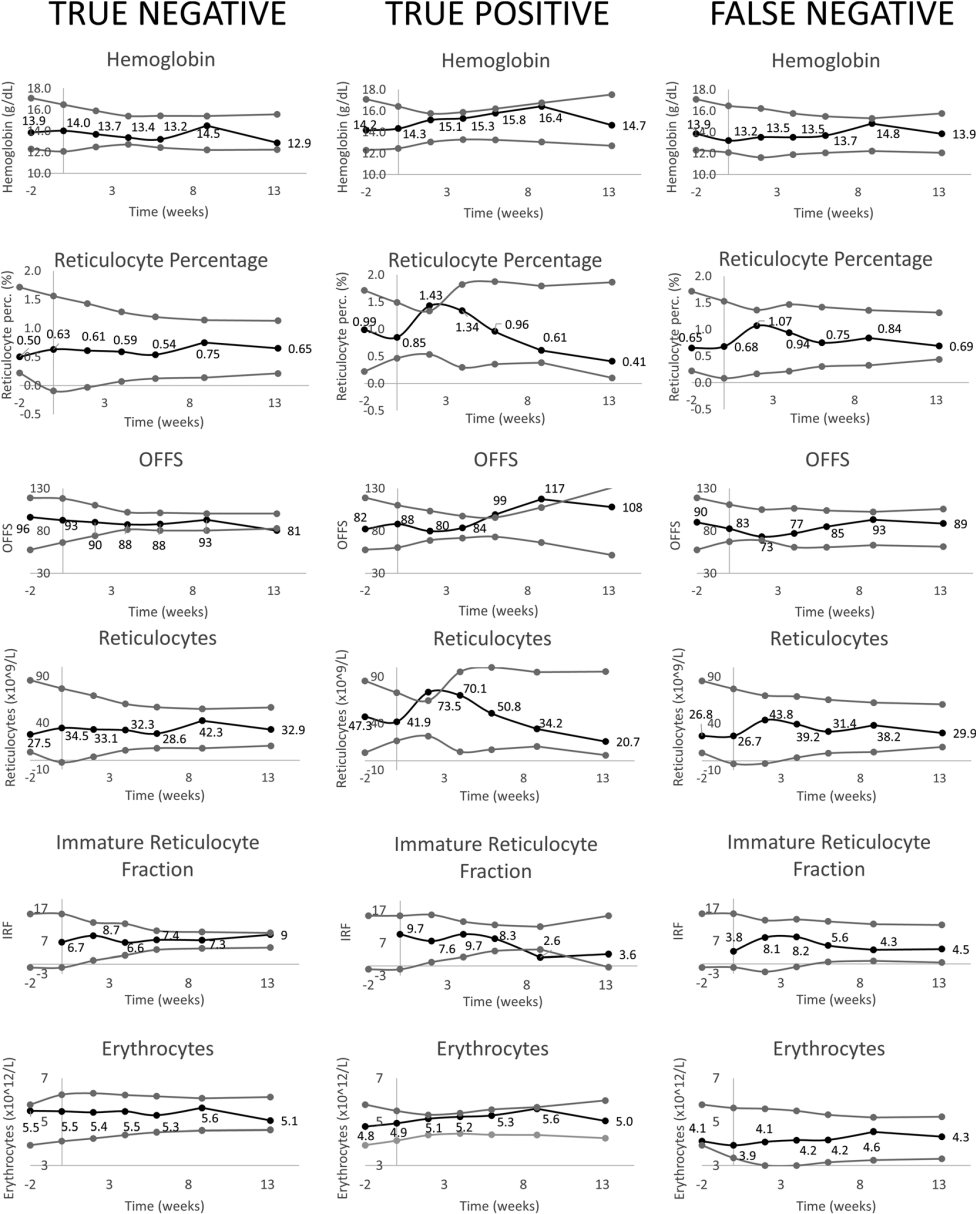
Athlete biological passport graphs exemplary subjects. Six main panels of the Athlete Biological Passport of three exemplary subjects. Black data points and the accompanying numbers are the values observed for this parameter in the particular subject. Grey points are the calculated 99.7% CI of the (individual) normal values calculated as described in the text. A, subject that received placebo that was correctly identified as not using rHuEPO. B, subject that received rHuEPO weekly for 8 weeks starting at time point 0 that was correctly identified as using rHuEPO. C, subject that received rHuEPO weekly for 8 weeks starting time point 0 that was incorrectly identified as not using rHuEPO. OFFS: Off‐score; IRF: Immature reticulocyte fraction.

A total of 47 ABPs based on the collected blood samples were rated for rHuEPO use by the two independent APMU staff members. Results of the ABP performance by Evaluator 1 and Evaluator 2 are depicted in Table 3. The sensitivity of the ABP by both evaluators was 91.3% and specificity was 100% for Evaluator 1 and 95.8% for Evaluator 2. Evaluator 1 classified 14 ABPs as positive, 7 as suspicious, and 26 as negative. Evaluator 2 classified 15 ABPs as positive, 7 as suspicious and 25 as negative. Of all 47 ABPs, 39 were scored identically by the evaluators. When the results for suspicious and positive were combined, 44 ABPs were scored identically. One discrepancy between evaluators was that Evaluator 2 designated an ABP of a placebo subject as suspicious, which was correctly designated as negative by Evaluator 1. Additionally, both evaluators designated a separate ABP as negative while it belonged to a subject from the rHuEPO group. Interestingly, the ABP from one rHuEPO‐treated subject was designated as negative by both evaluators. This ABP, together with a true positive and a true negative subject's ABP can be seen in Figure 3.

**Table 3:** Athlete biological passport performance characteristics (total *N* = 47)

**Measure**	**Evaluator 1**	**Evaluator 2**
	**Value (%)**	**Value (%)**
Sensitivity	91.3	91.3
Specificity	100.0	95.8
False negative rate	8.7	8.7
False positive rate	0.0	4.2
Precision	100.0	95.5
False discovery rate	0.0	4.5
False omission rate	7.7	8.0
Negative predictive value	92.3	92.0

## 7 DISCUSSION

The present study evaluated the performance of current doping detection methods for epoetin β (NeoRecormon) use by athletes. The sarcosyl‐PAGE urine assay based on 330 samples was shown to be highly sensitive for the applied dose of 5000 IU within a small time range of 2−6 days after dosing. Of all non‐rHuEPO samples 4.3% was labeled screening positive, but after the standard confirmation analysis no false positives were found. Findings for IEF showed a slightly lower sensitivity and slightly higher false presumptive finding rate of 6%. Evaluation of the ABP based on hematology samples had high sensitivity of 91.3% for rHuEPO use. Specificity was also high with 100% and 95.8% for the two evaluators. However, this research brought forward some concerns of all three methods.

### 7.1 Sarcosyl‐PAGE urine assay performance

The sarcosyl‐PAGE assay performed well 2−6 days after dosing, but outside this window sensitivity falls rapidly. Given the long‐lasting hematological effect of rHuEPO (red blood cell lifespan is around 120 days^21^), high frequency testing and intelligent testing strategies (eg, targeted testing in combination with the ABP) are necessary to attain a high probability to catch athletes abusing rHuEPO. A second concern with this assay is that a small part of the samples (5%) were non‐detectable, especially because this result occurred more often in rHuEPO treated subjects (83% of these samples). Non‐detectable results in general have been associated with variety of reasons. Degradation of EPO via neuraminidase activity, with consecutive loss of sialic acid moieties will result in a shift in molecular weight of EPO to lower molecular weight sections.^22^ Protease activity might lead to the disappearance of EPO. Of course suppression of endogenous production due to a negative feedback mechanism will also lower endogenous EPO levels, potentially below the limit of detection (LOD) of the method. Finally, urine dilution could disturb the assay.^23^ With regard to dilution however, only four samples with a non‐detectable result (24% of all non‐detectable samples) were strongly diluted, having an osmolality of <180 mOsm/kg (approximately corresponding to a specific gravity of <1.004,^24^ the applied cut‐off for not accepting a sample in anti‐doping procedures). In contrast, 30 samples (10%) with similarly low osmolality had positive or negative assay results. Therefore osmolality (alone) does not seem to explain a non‐detectable result, and the cause for such a result remains unclear. This finding also questions the validity of the current process of rejecting samples based on specific gravity when used in relation to testing for rHuEPO use. Of the 17 samples designated as non‐detectable for the sarcosyl‐PAGE assay, 13 had the same results in IEF, indicating the problem is probably sample related. Given that only three subjects had more than one sample (namely two, and for one subject three) designated as non‐detectable, this effect does not seem to be subject‐related. On top of this unknown effect leading to non‐detectable results, athletes might engage actively in manipulative behavior to prevent detection of rHuEPO use. Implementing an additional immunoassay in the routine doping procedure for non‐detectable samples evaluating if overall erythropoietin levels are sufficiently present in such samples, would therefore help interpret the result. If the overall erythropoietin levels in the immunoassay are low or absent, the non‐detectable result could be explained by low concentrations of erythropoietin (or no erythropoietin). If this is not the case, there might be a different cause for the non‐detectable result that needs further attention. The analysis could for example also show if there was a potential issue with recovery, or cross‐reactivity with specific erythropoietin fragments due to degradation.

**Figure 4:**
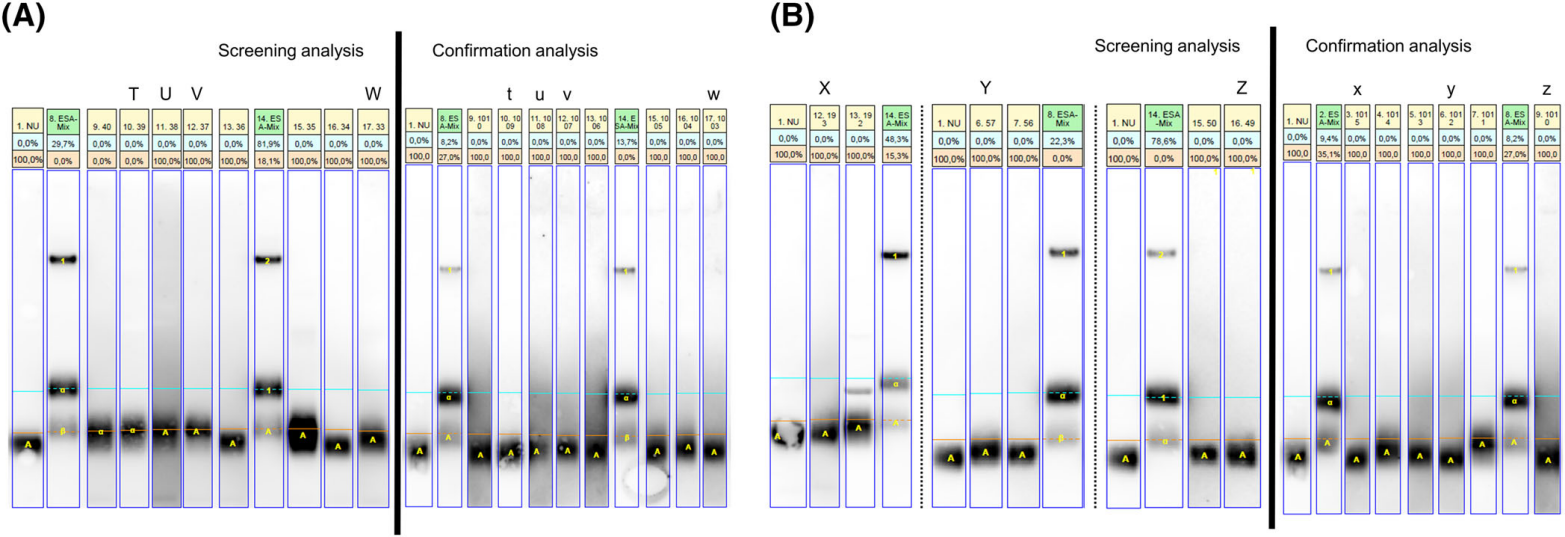
Sarcosyl‐PAGE urine assay results of screening and confirmation analysis of false presumptive findings. Images of the sarcosyl‐PAGE assay results taken from different gels with the nearest references (labeled ESA‐Mix) and the negative quality control (labeled NU) for each relevant lane. Letters indicate the relevant lanes, with the matching upper‐case and lower‐case letters indicating the same sample. The orange line indicates the level above which rHuEPO will stain. A**,** In lanes 10.39, 11.38, 12.37, and 17.33 urine samples taken from subjects receiving placebo were analyzed and determined as suspect for rHuEPO use. In lanes 10.1009, 11.1008, 12.1007, and 17.1003 the same samples are analyzed in the confirmation analysis. B, In lanes 12.193, 6.57, and 16.49 urine samples taken from subjects receiving placebo were analyzed and determined as suspect for rHuEPO use. In lanes 3.1015, 6.1012, and 9.1010 the same samples were analyzed in the confirmation analysis. All indicated samples in the screening analysis show staining above the orange line, which is what made them suspicious, with lanes 10.39, 11.38, and 12.37 even staining a large portion above the orange line. This is not the case in the same samples in the secondary analysis (compare with lanes 10.1009, 11.1008, 12.1007).

The aim of the screening procedure is to forward all potential positives and not to exclude any false negatives and in the confirmation procedure, this ‘philosophy is reversed’; ie, not to report any false positives, as described previously. The sarcosyl‐PAGE assay partly does allow for this at reasonable economically viable conditions with less than 5% false presumptive findings forwarded to confirmation. What is remarkable however, is that when the results from the same sample in the screening and confirmation analyses are compared, at least in some cases these are clearly different (Figure 4). Sample preparation errors (eg, sample swapping) cannot definitively be excluded as cause of these discrepancies, but given that false presumptive findings were observed in the screening analysis that was performed according to the standard procedure for sample preparation as for official doping samples, this seems unlikely. Furthermore, although no formal positive control samples were included in the confirmation analysis, authentic positive samples from the study samples were among the samples on all gels. In Figure 4 these are lanes 15.1005, 16.1004, 4.1014, and 7.1011 of the confirmation analysis gels and 9.40, 15.35, and 13.192 of the screening analysis gels and these can therefore serve as positive controls. Also, DTT and heating were used for all samples, and these processing steps were performed at the same time for all samples on a gel, making comparison between samples on one gel valid. Finally, degradation was not checked as this is not required per the WADA procedure for rHuEPO testing. However, such a factor would not explain the false presumptive finding in the screening analysis. In the absence of a convincing alternative explanation for the observed discrepancy in the outcome of the screening and confirmation procedure, variability in assay performance needs to be considered as an explanation as well.

**Figure 5:**
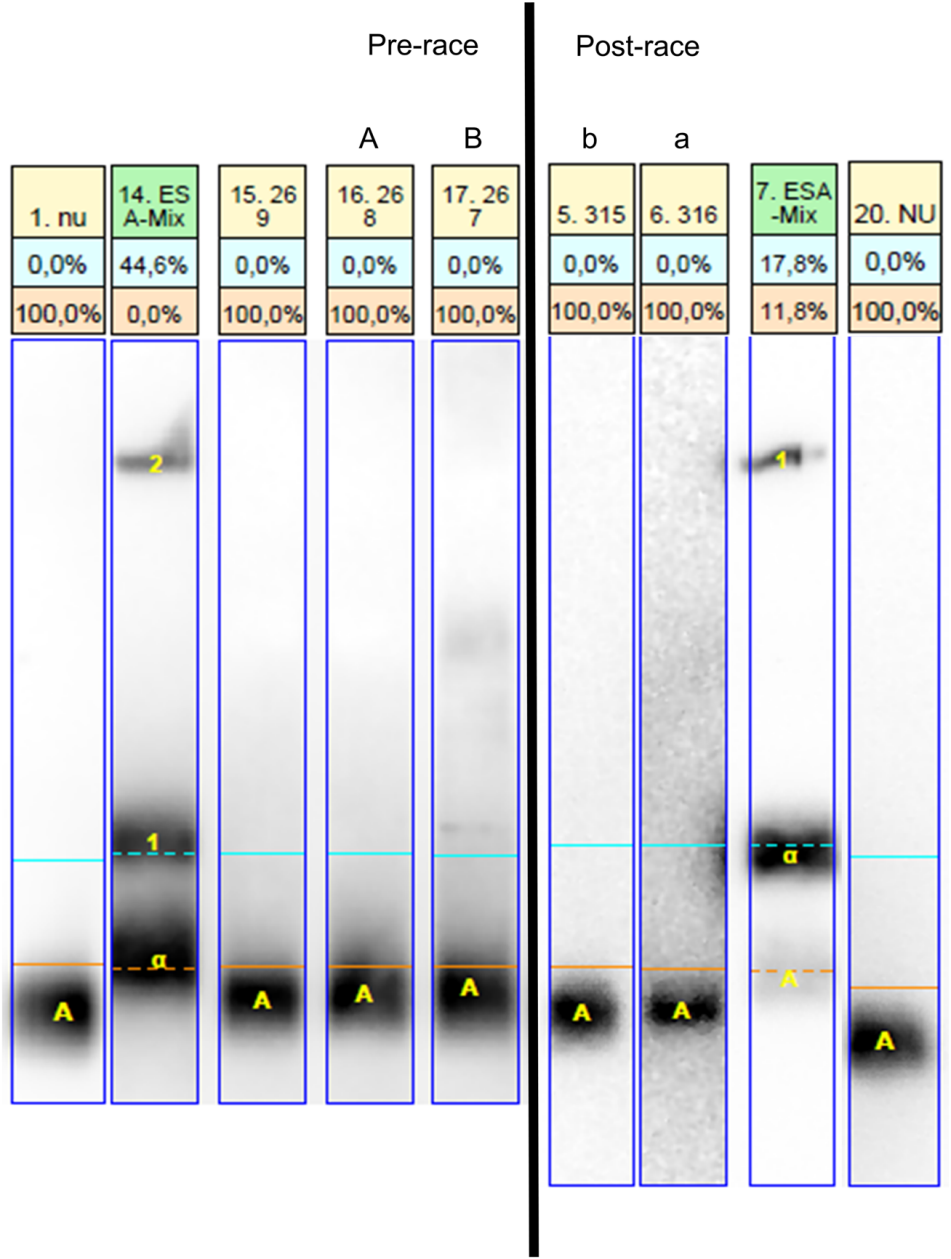
Example of changes in sarcosyl‐PAGE results during the race. Images of the sarcosyl‐PAGE assay results taken from two gels. In lanes 16.268 and 17.267, urine samples taken pre‐race from two subjects receiving rHuEPO were analyzed. In lanes 5.315 and 6.316, samples taken post‐race from the same subjects were analyzed. Letters indicate the relevant lanes, with A and a indicating lanes from one subject, and B and b from the other. Lanes labeled ESA‐Mix are positive controls. Lanes labeled NU are negative quality controls. The orange line indicates the level where rHuEPO will stain. In lanes 16.268 and 17.267, staining is clearly present above the orange line, while this is not the case in the samples from the same subjects that were taken only approximately 8 hours later, after the race, in lanes 5.315 and 6.316.

Finally, sensitivity of the assay did not seem to be affected by the maximal exercise test. The race however did seem to impact sensitivity, with six subjects that tested positive at days 11 and 12 before the race testing negative after the race. Of these six negative post‐race samples, two were non‐detectable, potentially due to effects of the exercise and hydration. The other four samples, however, were designated negative, indicating the race potentially affected the ability to detect rHuEPO. These four subjects went from positive to negative in approximately 8 hours, which is remarkable (Figure 5). One explanation could be that the concentration of erythropoietin in the post‐race samples was lower (possibly due to changes in urine concentration during the race), and that the faint signal that was detected pre‐race was therefore not visible post‐race.

### 7.2 Isoelectric focusing urine assay performance

Findings for the IEF assay were similar to the sarcosyl‐PAGE assay, although overall sensitivity slightly worse for IEF, which is similar to a previous study with micro‐doses of rHuEPO. The most striking difference between the two assays was a four‐fold higher incidence of samples not being detectable for the IEF (21%). This could be due to the fact that the IEF is not currently being used by the DoCoLab in Ghent and was only implemented for this study, and less routine led to more samples being designated as non‐detectable. Additionally, it could be due to the effect of previously described neuraminidase activity on IEF results. The LOD for sarcosyl‐PAGE as determined with reference standards was the same as for IEF. Samples were stored frozen immediately following collection until analysis. Samples were defrosted overnight in a fridge and aliquoted immediately before analysis. While for sarcosyl‐PAGE, mostly protease activity will lead to undetectable results and neuraminidase activity will lead to a shift (lower molecular weight) of the rHuEPO band, we believe that neuraminidase activity might lead to complete “disappearance” of the EPO bands in IEF and not in sarcosyl‐PAGE. This may contribute to the observed difference. Similar to the worrying finding for sarcosyl‐PAGE, the majority of the non‐detectable samples (75%) in IEF belonged to rHuEPO‐treated subjects. This higher incidence in samples from doped athletes in both IEF and sarcosyl‐PAGE indicates that, while the fact that no EPO can be detected at all is not a proof of doping, this information might be useful to plan target and/or follow up tests as rHuEPO use might be associated with a non‐detectable result. To rule out problems with the individual assay when such a result occurs, it could be useful to add a positive control to each sample, for example using EPO from a different animal species, as is common for most other doping analyses.

There was no overlap in false presumptive findings between the sarcosyl‐PAGE and IEF assays, indicating that such a result is not related to the sample.

In contrast to the sarcosyl‐PAGE assay, the maximal exercise test potentially did have an effect on the assay result of the IEF assay. Sensitivity was higher directly after the test compared to before. For the race, however, we observed a similar outcome as for the sarcosyl‐PAGE assay: three subjects tested positive before the race; after the race two tested negative, and one non‐detectable. The impairment in signal for these post‐race samples as observed in the sarcosyl‐PAGE assay (Figure 5) was not observed in the IEF assay for these two samples. However, this is solely based on observed signal intensities and not on a quantitative observation. As no internal standard was used, factors such as recovery differences in sample preparation between different batches/samples might also have played a role. If there indeed was an effect of exercise on the IEF test results, this effect was opposite for the race, compared to the maximal exercise test.

### 7.3 Athlete biological passport performance

In addition to evaluating the urine assays, we constructed an ABP so we could evaluate all tools used to detect rHuEPO use. This method had a high sensitivity of 91.3%. Given that this data is collected longitudinally and the method is less dependent on the time of sampling in relation to dosing compared to the urine assay, this approach not surprisingly has a better chance of detecting doping athletes. Nevertheless, almost 10% of subjects were not identified as suspicious or positive based on their ABP. See as an example the false negative subject in Figure 3, whose variation in hematological parameters is actually very similar to that of the placebo subject depicted in the left column of panels. This means that some athletes might not be at risk for detection based on the ABP despite using rHuEPO. Moreover, in practice, sensitivity of the ABP might fall due to potential lower doses being used (micro‐dosing) and less optimal timing of blood samples as the anti‐doping organization does not have the information on dosing times. Other studies evaluating the performance of the ABP showed somewhat ambiguous findings. One study used approximately half the weekly dose of our study after a high dose period of 250 IU/kg three times a week and found 100% subjects to have at least one suspicious or abnormal measurement.^25^ This difference with our findings could be due to the very high starting dose used. Additionally, this study did not investigate evaluator determination based on the ABP, but only whether individual values were outside the ABP reference ranges. Also remarkable is that a second, unblinded, study using doses building up to a similar dose to our weekly dose, found no measurements during the study being flagged as abnormal.^26^ This study used 99.9% likelihood ranges and did not investigate evaluator determination based on the ABP. Our own model used similar limits (99.7%) indicating the fact that an evaluator interpreted the ABP in our study might give more insight into the observed pattern.

### 7.4 Limitations

There are several limitations to our study. First, although unlikely, it cannot be ruled out that placebo‐treated subjects administered rHuEPO outside the proceedings of the study. Given that false presumptive findings were distributed over many subjects, this seems an unlikely explanation for these observations.

Second, for reasons of restrictions in time and personnel, confirmation analysis was not performed for isoelectric focusing, and only for a selection of 14 samples, including all false presumptive findings, for sarcosyl‐PAGE. In this respect, our study design did not follow regular anti‐doping procedures to forward all screening positive samples to a confirmation analysis, nor did it perform a third (B‐sample) analysis or solicit a second opinion by an expert. As the confirmation analysis only included a limited number of samples, the characteristics of this confirmation analysis by itself, including the false positive rate, is somewhat uncertain.

Furthermore, most subjects received higher doses (8000 or 10 000 IU) at the last dose and the accompanying urine samples taken more than 10 days after dosing, than at the second dose (5000 IU), which could have impacted the urine assay results over time. But even with a higher dose sensitivity was well below 40% for these samples. Additionally, it is not possible to verify if sample swapping in the preparation or re‐labeling of samples occurred, which might be an alternative explanation to the discrepancy observed for several samples between results for screening and confirmation analysis. However, this seems unlikely as the screening assay, which showed false presumptive findings, used the standard sample preparation procedure as for official doping samples. Moreover, such events would then also contribute to overall assay performance in the official WADA procedure. The re‐labeling procedure performed here between the screening and confirmation analysis is not part of the official WADA procedure, but was also performed by double‐checking by two staff members. Finally, observed characteristics for the assays apply to the materials and methods used here. Deviations, such as applying a 1.0 mm gel in contrast to the 1.5 mm gel used in this study (as based on a previous publication^18^), might change the assay performance to some extent.

For the ABP, a limitation was that samples were analyzed on a different type of analyzer, although from the same manufacturer, than the Sysmex XT 2000i that is used by anti‐doping laboratories. In addition, the ABP algorithm used by WADA was not available to us, and so the current method was an approximation of the official procedure. Nevertheless, these discrepancies should not have a major impact on the ABP profiles and so the outcome of the review of the ABPs, we feel, can be considered indicative of the first stage of ABP performance at the APMU.

Information about factors that might influence markers in the ABP (eg, altitude training) would be available for the evaluator assessment. This information was not systematically recorded in the present study, and although no such events were reported by subjects, occurrence could have impacted ABP review. It should also be noted that the relatively high sampling frequency applied in this study, with intervals of as short as two weeks, could lead to the individual reference ranges moving along with the observed values more than with longer intervals and thereby reducing the chance values to fall outside the range. The selected window is however the smallest acceptable window according to the ABP and so these data should still be correctly interpretable. Finally, in the WADA setting, after assessment of an ABP by an APMU, suspicious ABPs will be sent for review to a WADA hematology expert. If this expert confirms the suspicious finding, they and two other experts will determine, based on all available information and possibly additional requested information, whether the profile is indeed indicative of blood doping. For this study, several WADA hematology experts were approached to participate in assessment of the ABPs of this study, but all of them declined. Therefore, the results reported here can only be considered to resemble the first part of the official system. But as only suspicious results are forwarded to the expert, ABP sensitivity would at best stay at the same level.

## 8 CONCLUSION

The sarcosyl‐PAGE urine assay for rHuEPO (specifically epoetin β) did not show false positive results after confirmation analysis, but it does have shortcomings such as a limited detection window. In addition, it is of some concern that in this study we observed a higher rate of non‐detectable samples in subjects treated with rHuEPO. Adding a control internal standard to each sample and measuring total erythropoietin levels using an immunoassay could address the latter issue. The IEF assay in our study was inferior to the sarcosyl‐PAGE assay. The ABP had a much higher sensitivity than the assays using a single urine sample, although it too did not classify all passports correctly. In summary, we showed that all three methods evaluated in this study have their shortcomings and challenges and that it is critical to continue research to improve existing and develop new doping detection methods.

### KEY FINDINGS

World Anti‐Doping Agency methods for detection of recombinant human erythropoietin were evaluated in trained cyclists. Detection in urine by sarcosyl‐PAGE had a sensitivity of 63.8% and isoelectric focusing of 58.6%, with a peak in sensitivity between 2 and 6 days after dosing, rapidly dropping outside this window. False presumptive finding rates were 4.3% and 6%, respectively, but none of the false presumptive findings tested positive in the confirmation analysis. Sensitivity of the athlete biological passport that integrates longitudinal hematological data was 91.3%.

## CONTRIBUTORS

JH, PvE, JR, PG, FS, JD, HR, OdH, JB, and AC were involved in the study design. JH, PvE, PG, FS, JB, and AC were involved in the execution of the study. JH, JR, PG, HR, and OdH were involved in participant recruitment. JH wrote the first draft and final manuscript with input from PvE, JR, PG, FS, TP, JD, OdH, JB and AC. PvE was in charge of the urine analysis and interpretation of the results.

## DECLARATION ON INTEREST

We declare no competing interests.
